# Let-7i-5p enhances cell proliferation, migration and invasion of ccRCC by targeting HABP4

**DOI:** 10.1186/s12894-021-00820-9

**Published:** 2021-03-28

**Authors:** Yujie Liu, Xing Hu, Liang Hu, Changjing Xu, Xuemei Liang

**Affiliations:** 1 Department of Geriatric Medicine, The Affiliated Hospital of Southwest Medical University, Luzhou, 646000 China; 2 Department of General Practice, The Affiliated Hospital of Southwest Medical University, Luzhou, 646000 China; 3 Department of Cardiovascular Surgery, The General Hospital of Western Theater Command, Chengdu, 610083 China; 4 Department of Pharmacy, The Affiliated Hospital of Southwest Medical University, Luzhou, 646000 China

**Keywords:** Clear cell renal cell carcinoma, Let-7i-5p, HABP4, Cell proliferation, Cell migration, Cell invasion

## Abstract

**Background:**

Clear cell renal cell carcinoma (ccRCC) is one of the best-characterized and most pervasive renal cancers. The present study aimed to explore the effects and potential mechanisms of let-7i-5p in ccRCC cells.

**Methods:**

Using bioinformatics analyses, we investigated the expression of let-7i-5p in The Cancer Genome Atlas (TCGA) database and predicted biological functions and possible target genes of let-7i-5p in ccRCC cells. Cell proliferation assay, wound healing assay and transwell invasion assay were conducted to characterize the effects of let-7i-5p in ccRCC cells. To verify the interactions between let-7i-5p and HABP4, dual-luciferase reporter assay, quantitative real-time polymerase chain reaction, and western blotting were conducted. Rescue experiments were used to investigate the relationship between let-7i-5p and HABP4.

**Results:**

TCGA data analysis revealed that ccRCC tissues had significantly increased let-7i-5p expression, which was robustly associated with poor overall survival. Further verification showed that ccRCC cell proliferation, migration and invasion were inhibited by let-7i-5p inhibitor but enhanced by let-7i-5p mimics. Subsequently, HABP4 was predicted to be the target gene of let-7i-5p. TCGA data showed that ccRCC tissues had decreased expression of HABP4 and that HABP4 expression was negatively correlated with let-7i-5p. Further verification showed that downregulation of HABP4 expression promoted cell proliferation, migration and invasion. The dual-luciferase reporter gene assay suggested that the let-7i-5p/HABP4 axis was responsible for the development of ccRCC.

**Conclusion:**

Our results provide evidence that let-7i-5p functions as a tumor promoter in ccRCC and facilitates cell proliferation, migration and invasion by targeting HABP4. These results clarify the pathogenesis of ccRCC and offer a potential target for its treatment.

**Supplementary Information:**

The online version contains supplementary material available at 10.1186/s12894-021-00820-9.

## Background

Renal cell carcinoma (RCC), one of the most common urological tumors, can be categorized into non-clear cell renal cell carcinoma (nccRCC) and clear cell renal cell carcinoma (ccRCC) based on cytogenetic and histological signatures [1, 2]. ccRCC is the most common type of RCC, accounting for 70% of cases [3]. The early signs and symptoms of ccRCC are challenging to distinguish, with most patients already having middle-to-late stage ccRCC upon diagnosis [4].

Given that most patients with ccRCC are not sensitive to chemotherapy or radiation, surgical treatment has remained most effective [5, 6]. Thus, exploring the molecular mechanisms underlying the development of ccRCC and identifying effective treatment strategies to improve the survival rate of patients with ccRCC hold considerable clinical value.

MicroRNAs (miRNAs) are a class of endogenous, small non-protein coding single-stranded RNA molecules that regulate gene expression by binding to messenger RNA (mRNA) and inhibiting mRNA translation or promoting its degradation [7, 8]. Let-7, one of the first miRNAs to be discovered, has been found to be highly conserved and widely expressed among species [9, 10]. As a member of the let-7 family, let-7i-5p has been shown to play an important role in the proliferation and metastasis of various tumors [11–13]. However, the role of let-7i-5p in ccRCC has yet to be studied.

Hyaluronan-binding protein 4 (HABP4, also named Ki-1/57), an intracellular cross-reactant of the monoclonal antibody Ki-1, has been the first protein used to specifically detect malignant cells in Hodgkin’s lymphoma [14]. It is involved in gene expression regulation at both the transcriptional and mRNA metabolism levels. Studies in transcriptome data have suggested that HABP4 can function as a tumor suppressor gene by affecting the expression of genes involved in cell proliferation, cell cycle, and apoptosis [15]. However, the biological effects of HABP4 in ccRCC have yet to be elucidated.

The present study utilized The Cancer Genome Atlas (TCGA) database to study the prognostic significance of let-7i-5p expression levels in ccRCC, as well as investigate the effects of let-7i-5p on ccRCC proliferation and metastasis. Furthermore, this study explored the function of HABP4 in ccRCC and the negative regulatory relationship between let-7i-5p and HABP4 to further our understanding of the molecular mechanism underlying ccRCC tumorigenesis and offer a potential target for ccRCC therapies.

## Methods

### Retrieving ccRCC data from TCGA

The level 3 data on ccRCC were obtained from in TCGA using the UCSC Xena Browser (https://xenabrowser.net/). Using RNAseq (IlluminaHiseq), let-7i-5p expression was measured in 70 normal samples and 494 primary ccRCC tumor samples, whereas HABP4 expression was measured in 72 normal samples and 533 primary ccRCC tumors. The clinicopathological data of the patients with intact survival information, including age at initial pathologic diagnosis, histology, tumor grade, clinical stage, recurrence status, and living status, were downloaded for survival-related analysis.

### Bioinformatics analysis of possible let-7i-5p targets and genes negatively related to let-7 expression in ccRCC

Possible targets of miR-361-3p were predicted using DIANA (http://www.microrna.gr/microT-CDS), miRanda (http://www.microrna.org/microrna/home.do), TargetScan (http://www.targetscan.org/vert_72/), and miRmap (https://mirmap.ezlab.org/app/). Moreover, genes negatively correlated with let-7i-5p in TCGA-CESC were identified using LinkedOmics (http://www.linkedomics.org/login.php). The overlapping subset between the negatively correlated genes and predicted target genes was identified using Venn diagram analysis.

### Cell culture

Human ccRCC cell lines 786-0 and 769-p were purchased from Cell Bank (Chinese Academy of Sciences, Shanghai, China). All cell lines were cultured in Dulbecco’s Modified Eagle’s Medium (DMEM) (HyClone, Thermo, USA) supplemented with 10% fetal bovine serum, 100 U/mL penicillin, and 100 mg/mL streptomycin and subsequently incubated at 37℃ in a 5% CO_2_ atmosphere.

### Cell transfection

The mimics NC, inhibitor NC, siControl, let-7i-5p mimics, let-7i-5p inhibitor, siHABP4#1 and siHABP4#2 were designed and provided by Guangzhou RiboBio Co., Ltd., (Guangzhou, China). After reaching 70% confluence, cells were transfected or co-transfected with Lipofectamine 3000 (Invitrogen, Carlsbad, CA, USA) according to the manufacturer’s instructions. Quantitative real-time polymerase chain reaction (qRT-PCR) and western blotting were then used to verify the transfection efficiency. After transfection for 48 h, ccRCC cells were collected for subsequent experiments. The sequences of miRNA mimics and miRNA inhibitor were as follows: hsa-let-7i-5p mimics, sense: 5′-UGAGGUAGUAGUUUGUGCUGUU-3′, antisense: 5′-CAGCACAAACUACUACCUCAUU-3′; miRNA mimics negative control (miRNA mimics NC), sense: 5′-UUCUCCGAACGUGUCACGUTT-3′, antisense: 5′-AGGUGACACGUUCGGAGAATT-3′; hsa-let-7i-5p inhibitor: 5′-AACAGCACAAACUACUACCUCA-3′; miRNA inhibitor NC: 5′-CAGUACUUUUGUGUAGUACAA-3′. The siRNA sequences were as follows: siHABP4 #1, sense: 5′-AUCCCAGCUGGAGAUUAAUUUTT-3′ and antisense 5′-AAAUUAAUCUCCAGCUGGGAUTT-3′; siHABP4 #2, sense: 5′-CAAGUUCAAGAGAUGACUUUATT-3′ and antisense 5′-UAAAGUCAUCUCUUGAACUUGTT-3'.

### RNA extraction and qRT‑PCR analysis

Total RNA was extracted from the ccRCC cell lines using Trizol Universal reagent according to the manufacturer’s instructions. The cDNA template used in mRNA qPCR was synthesized using reverse transcriptase and oligo (dT) primer (Promega). qRT‑PCR was conducted on an ABI 7500 Sequence Detection System (Life Technologies, USA) using the corresponding PCR reagent according to the manufacturer’s instructions. The primers used were as follows: HABP4, 5′-AAGAGCTGAGCGGAGATCCTAC-3′ (forward) and 5′-TCCTCTCAACGGTCTGTCTCGA-3′ (reverse); GAPDH, 5′-GAAGGTGAAGGTCGGAGTC-3′ (forward) and 5′-GAAGATGGTGATGGGATTTC-3′ (reverse). cDNA template used in miRNA qPCR was synthesized using the miScript II Reverse Transcription Kit (Qiagen, Germany) according to the manufacturer’s protocol. Quantification of let-7i-5p was carried out by miScript Primer Assay (Hs_let-7i_1 miScript Primer Assay MS00003157 Qiagen, Germany), normalizing over RNU6B control (Hs_RNU6-2_11 miScript Primer Assay MS00033740 Qiagen, Germany) and using the miScript SYBR Green PCR kit (Qiagen, Germany) on ABI 7500 Sequence Detection System (Life Technologies, USA). RNU6B and GAPDH served as internal controls. Relative expression was determined using the 2^−∆∆CT^ method.

### Protein extraction and western blot analysis

After cells were trypsinized (0.05% Trypsin EDTA, Invitrogen), cell pellets were lysed with RIPA buffer (Beyotime, Shanghai, China) and kept in ice for 30 min with intermittent vortexing. The lysate was obtained through centrifugation at 12,000 rpm for 20 min at 4℃, after which its protein concentration was determined using BCA protein assay reagents (Beyotime, Shanghai, China). Protein samples were fractionated in a 10% sodium dodecyl sulfate–polyacrylamide gel and transferred to a polyvinylidene difluoride (PVDF) membrane (Immobilon-P transfer membrane, Millipore, Saint-Quentin-en-Yvelines, France). Membranes were blocked with 5% non-fat milk and washed with Tris-Buffered Saline and Tween 20 (TBST) and then incubated with primary antibody (anti-HABP4, Abnova, Taipei, Taiwan; anti-GAPDH, Proteintech, Wuhan, China) at 4℃ for 24 h. The PVDF membranes were extensively washed three times and then incubated with horseradish peroxidase-conjugated secondary antibody. After three washes with TBST, the membranes were detected using the ECL detection kit (Beyotime, Shanghai, China). GAPDH was used as an internal control. The integrated optical density (IOD) of the individual bands on blots was measured by ImageJ and the ratio of HABP4 protein to the housekeeping protein GAPDH was calculated.

### Dual-luciferase reporter assay

The 3′-untranslated region (UTR) of HABP4 was synthesized, annealed, and inserted into the pmirGLO luciferase reporter vector (Promega Corporation, Madison, WI, USA). The 3′-UTR wild-type (WT) HABP4 sequence (ACTACCTC) complementary to the binding site of let-7i-5p was replaced with a mutated (MUT) HABP4 sequence (ACATGGTC). Let-7i-5p mimics were co-transfected with pmirGLO-WT-HABP4 or pmirGLO-MUT-HABP4 using Lipofectamine 3000 (Invitrogen, Carlsbad, CA, USA). After the cells were incubated for 48 h, luciferase activity was measured using the dual-luciferase reporter assay kit (Beyotime, Shanghai, China).

### MTT assay

Transfected cells were seeded at 2.0 × 10^3^ cells/well in 96-well plates. Subsequently, 10 μL of 5 mg/ml MTT (Beyotime, Shanghai, China) was added into each of the 96 wells for 4 h at 37℃, after which 100 μL DMSO was added to dissolve the purple crystals. Absorbance was determined using a spectrophotometer at 570 nm.

### Wound healing assay

Transfected cells were seeded on 6-well plates with a fresh medium containing 10% FBS.

After forming a monolayer culture, cells were scratched using a sterile 200 µL pipette. The cells were washed twice with PBS, after which the medium was replaced with a serum-free medium. Images of the wound were taken on day 0 and after 24 h. The wound healing rate was calculated using the formula wound healing rate (%) = [(width at 0 h − width at 24 h)/(width at 0 h)] × 100.

### Transwell invasion assay

Cells were seeded in the upper compartment of transwell chambers (Corning, NY, USA) that were pre-coated with Matrigel (BD Biosciences, CA, USA). DMEM containing 20% FBS was added to the bottom compartments as a chemoattractant. After 24 h, the non-invading cells were wiped out, whereas the invading cells on the lower surface were fixed with 4% paraformaldehyde and stained with crystal violet. Images were obtained using an Olympus microscope.

### Statistical analysis

Statistical analysis was conducted using GraphPad Prism 6.0 (GraphPad Inc., La Jolla, CA, USA) or SPSS 23.0 software package (SPSS Inc., Chicago, IL, USA). Values were expressed as mean ± SD, with *P* values < 0.05 indicating statistical significance.

## Results

### High let-7i-5p expression was correlated with poor patient prognosis

**Fig. 1:**
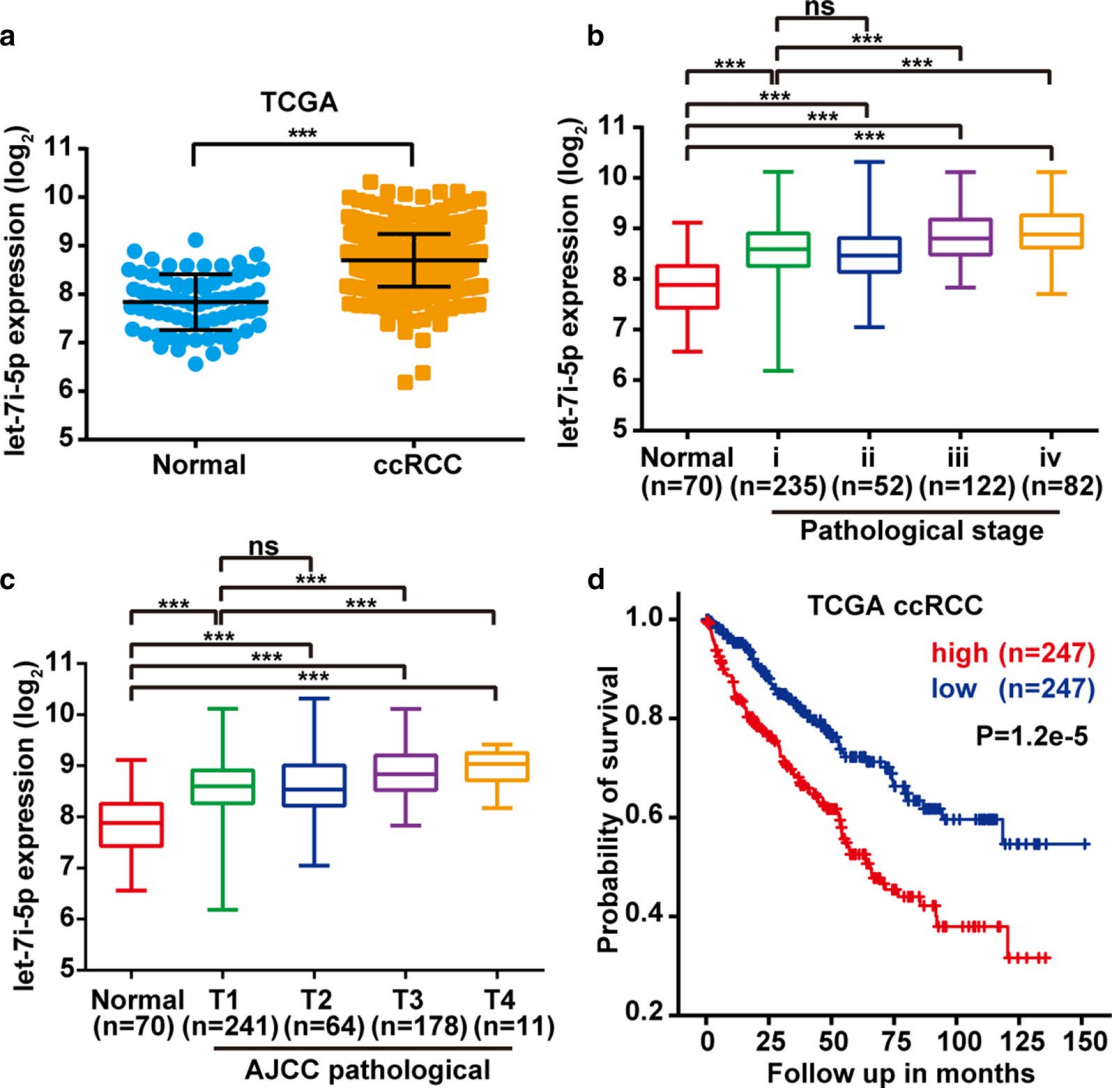
Correlation between let-7i-5p expression and ccRCC pathological information. **a** The relative expression of let-7i-5p in TCGA dataset. **b**, **c** The relative expression of let-7i-5p across different pathological stages and AJCC pathological T stage. **d** Kaplan–Meier analysis of overall survival according to high or low let-7i-5p expression in the TCGA database. Data are presented as mean ± SD. ns > 0.05, *P < 0.05; **P < 0.01; ***P < 0.001

To determine whether let-7i-5p expression was a prognostic factor for poor survival, expression data from the TCGA dataset were analyzed. Accordingly, ccRCC tissues had a higher let-7i-5p expression level than normal kidney tissues (Fig. 1a). Furthermore, we explored the relationship between let-7i-5p expression level and clinicopathological characteristics, with the results summarized in Table 1. Our results showed a significant difference between pathological stage, American Joint Committee on Cancer (AJCC) T, N, M stages, and tumor status. Meanwhile, the level of let-7i-5p expression increased significantly in the pathological stage (III and IV) and AJCC stages (T3 and T4) (Fig. 1b, 1c, Additional file 1: Table S1). Given that the patients with different survival outcomes had different levels of expression, we decided to explore whether let-7i-5p was a prognostic marker in ccRCC. Accordingly, Kaplan–Meier analysis showed that high let-7i-5p expression was associated with poor prognosis (Fig. 1d). These data indicated that let-7i-5p might play an oncogenic role in ccRCC.

**Table 1:** Correlation between let-7i-5p expression and clinicopathological variables of ccRCC using The Cancer Genome Atlas datasets

Clinicopathological features	Cases	Let-7i-5p expression		*t*	*p*
		Low	High		
Group					
ccRCC	494	247	247	− 12.313	0.000
Normal	70	66	4		
Age					
≥ 60	265	125	140	− 1.502	0.134
< 60	229	122	107		
Gender					
Male	320	155	165	− 0.986	0.325
Female	174	92	82		
Race					
White	426	210	216	− 1.348	0.178
Black or African American	53	30	23		
Pathological stage					
III–IV	204	76	128	− 6.306	0.000
I–II	287	171	116		
AJCC pathological T					
T3–T4	189	69	120	− 5.892	0.000
T1–T2	305	178	127		
AJCC pathological N					
N1	17	3	14	− 2.843	0.005
N0	220	109	111		
AJCC pathological M					
M1	78	23	55	− 4.048	0.000
M0	387	209	178		
Tumor status					
With tumor	144	51	93	− 4.807	0.000
Tumor-free	308	178	130		
Hemoglobin					
Low	244	109	135	− 1.898	0.058
Normal	175	98	77		

### Let-7i-5p enhanced cell proliferation, migration and invasion of ccRCC

**Fig. 2:**
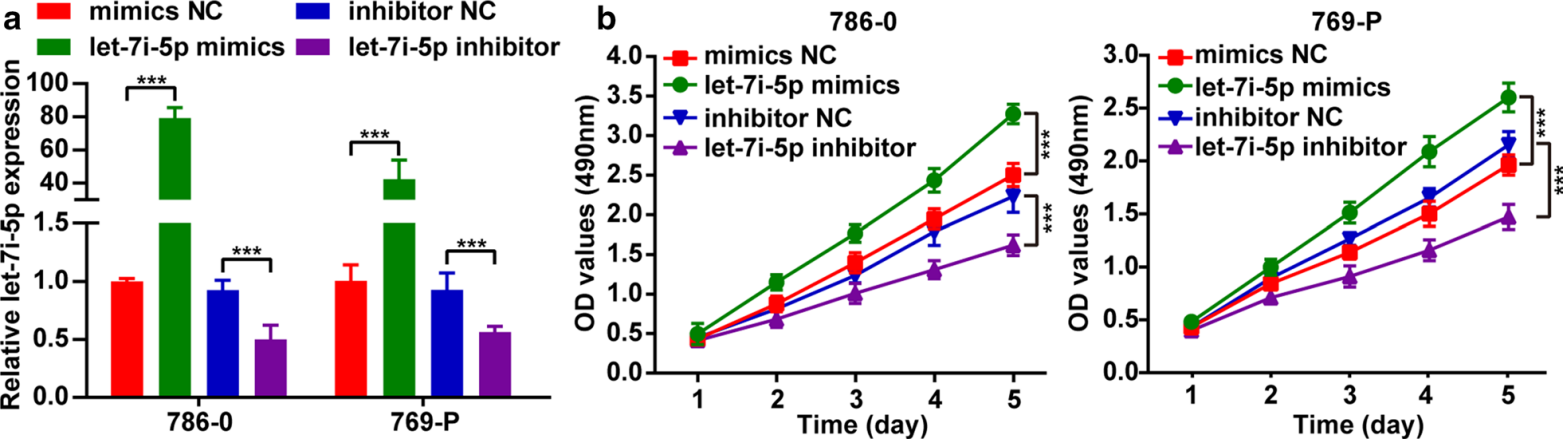
The regulatory effect of let-7i-5p on ccRCC cell proliferation. **a** After transfecting 786-0 and 769-P cells with the let-7i-5p mimic or inhibitor, the expression of let-7i-5p was detected using qRT-PCR. **b** MTT assays were used to elucidate the effects of let-7i-5p on cell proliferation. Data are presented as mean ± SD. ***P < 0.001

**Fig. 3:**
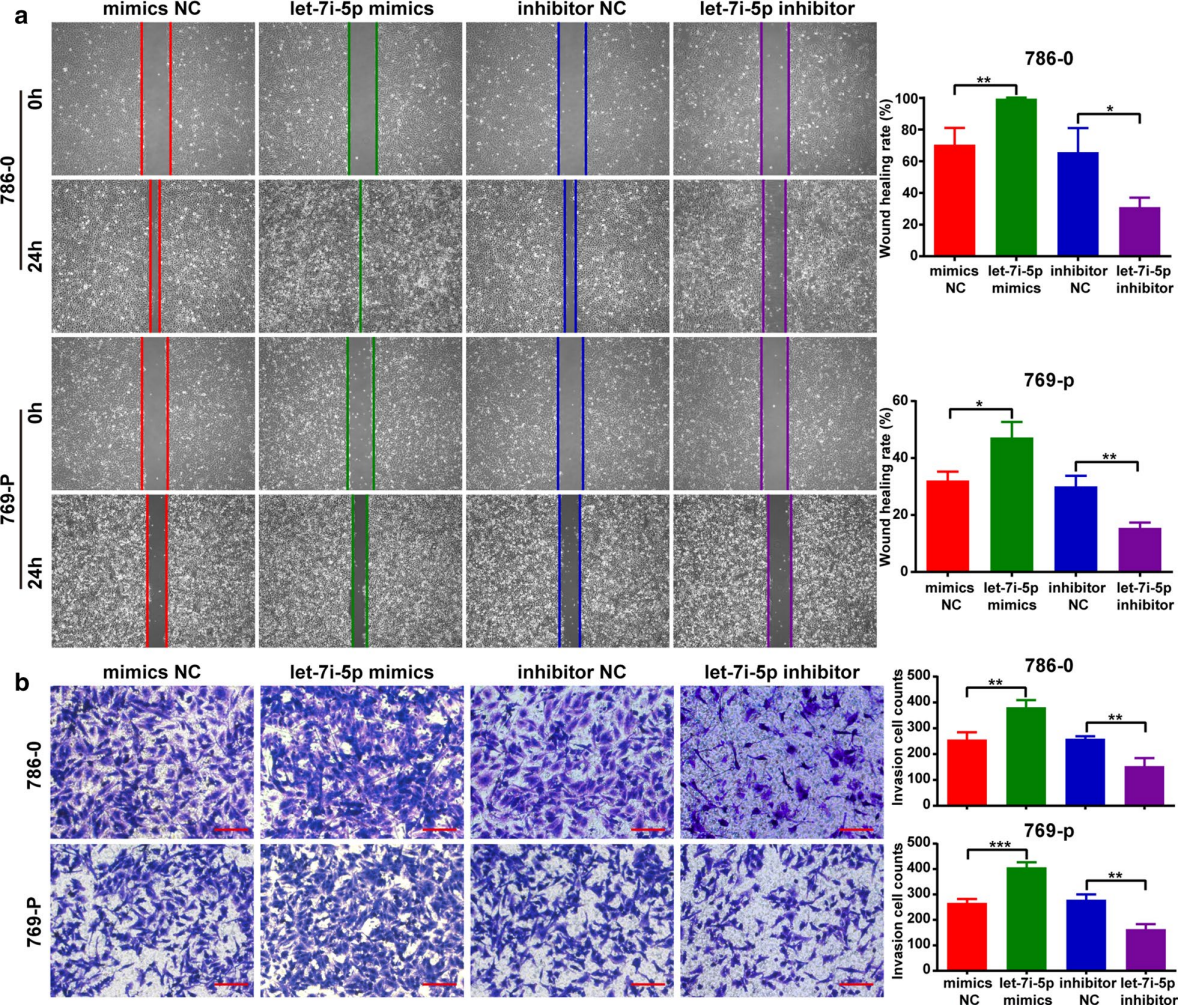
The regulatory effect of let-7i-5p on ccRCC cell migration and invasion. **a** Wound healing assays were used to elucidate the effects of let-7i-5p on cell migration. **b** Transwell invasion assays were used to analyze the role of let-7i-5p on cell invasion. Scale bar = 100 μm. Data are presented as mean ± SD. *P < 0.05; **P < 0.01; ***P < 0.001

Two kidney cancer cell lines (786-0 and 769-P) were used to examine the function of let-7i-5p in ccRCC. Accordingly, transfecting a let-7i-5p mimic into 786-0 and 769-P cells upregulated the expression of let-7i-5p, whereas transfecting a let-7i-5p inhibitor downregulated the same (Fig. 2a). MTT assay results showed that let-7i-5p overexpression could promote cell proliferation, whereas let-7i-5p downregulation could inhibit cell proliferation (Fig. 2b). Furthermore, a wound healing assay and transwell invasion were used to analyze the effects of let-7i-5p on ccRCC cell migration and invasion, respectively. Accordingly, we found that let-7i-5p overexpression could promote cell migration and invasion, whereas let-7i-5p downregulation could inhibit cell migration and invasion (Fig. 3a, b). Collectively, our results suggested that let-7i-5p played a facilitative role in ccRCC cell proliferation, migration, and invasion.

### Predicted target genes of let-7i-5p

**Fig. 4:**
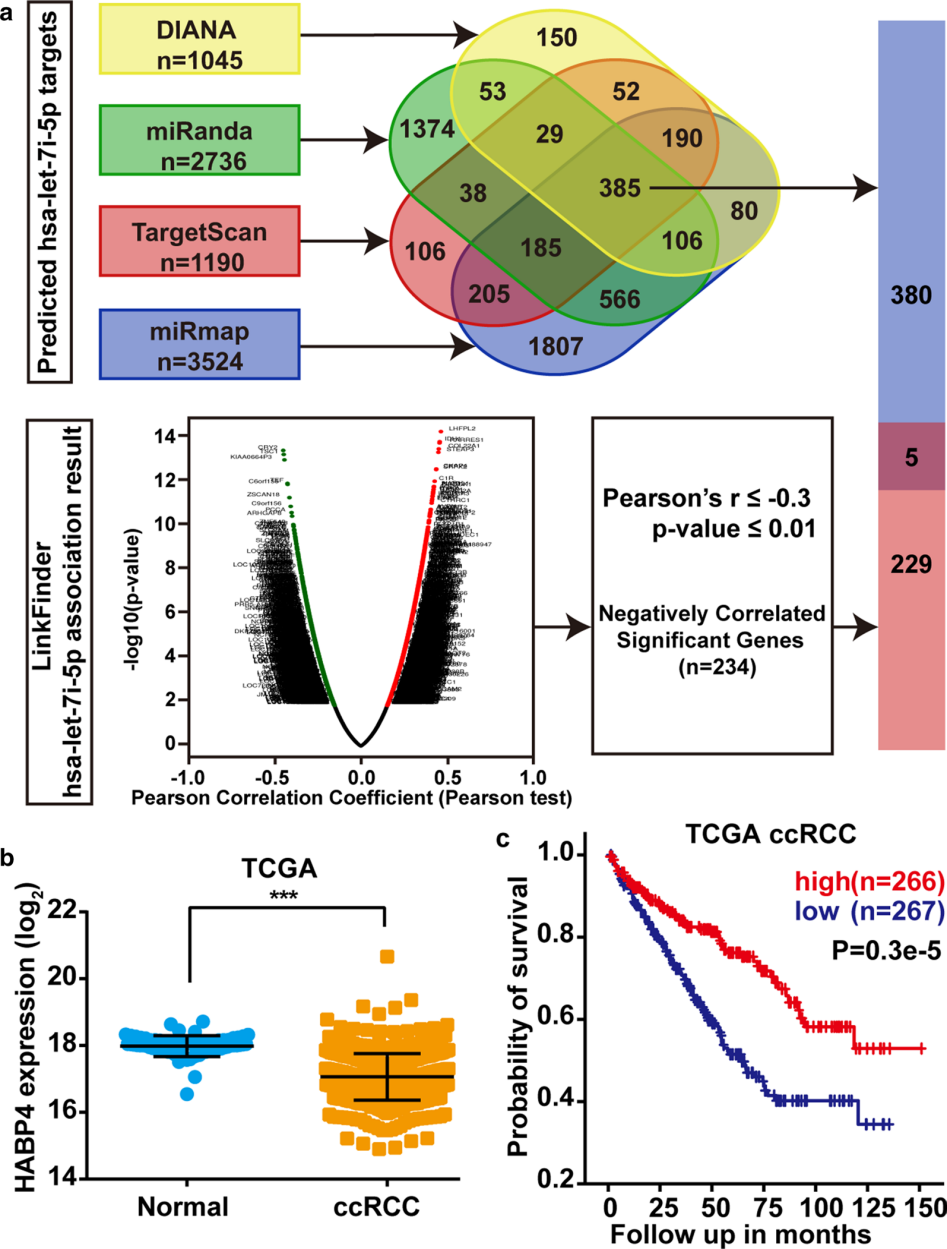
Predicted target genes of let-7i-5p. **a** Four different miRNA-target prediction programs (DIANA, miRanda, TargetScan, and miRmap) were used to predict putative target genes of let-7i-5p. LinkedOmics database was used to analyze the negative correlation between let-7i-5p and mRNA expression. **b** The relative expression of HABP4 in the TCGA dataset. **c** Kaplan–Meier analysis of overall survival according to high or low HABP4 expression in the TCGA database. Data are presented as mean ± SD. ns > 0.05, **P < 0.01; ***P < 0.001

To explore the potential molecular mechanisms through which let-7i-5p promotes ccRCC cell proliferation, migration, and invasion, we used four different miRNA-target prediction programs (DIANA, miRanda, TargetScan, and miRmap) to predict the putative target genes of let-7i-5p. A total of 385 putative miRNA-target genes were predicted by DIANA, miRanda, TargetScan, and miRmap programs (Fig. 4a). Using LinkedOmics to analyze mRNA/miRNA expression data, we identified 234 genes that negatively correlated with let-7i-5p expression (Fig. 4a, Additional file 2: Table S2). Furthermore, five genes were obtained after comparing these two groups of genes. Statistical analysis of the binding score and the association between the five genes and let-7i-5p found that HABP4 had a high binding score in four different miRNA-target prediction programs (Table 2). To determine the function of HABP4 in ccRCC, the expression and prognosis of HABP4 were analyzed using the TCGA database. Accordingly, TCGA data showed that ccRCC tissues had lower HABP4 expression than normal kidney tissues (Fig. 4b). Moreover, Kaplan–Meier analysis showed that low HABP4 expression was associated with poor prognosis (Fig. 4c). Overall, our results suggested that HABP4 may be a novel target gene of let-7i-5p.

**Table 2:** Statistical analysis of the binding score and association between the five genes and let-7i-5p

Target	Binding score				Association	
	miRmap	TargetScan	miRanda	DIANA	Pearson’s r	p-value
HABP4	84.7	− 0.35	− 0.75	0.83	− 0.33	5.82E−08
CPEB3	83.7	− 0.19	− 0.21	0.83	− 0.30	9.47E−07
TSC1	79.6	− 0.30	− 0.35	0.82	− 0.45	5.52E−14
SLC25A4	62.0	− 0.33	− 0.11	0.71	− 0.38	6.06E −10
PPARGC1A	18.6	− 0.18	− 0.11	0.96	− 0.30	9.18E −07

### Downregulation of HABP4 expression promoted cell proliferation, migration and invasion

**Fig. 5:**
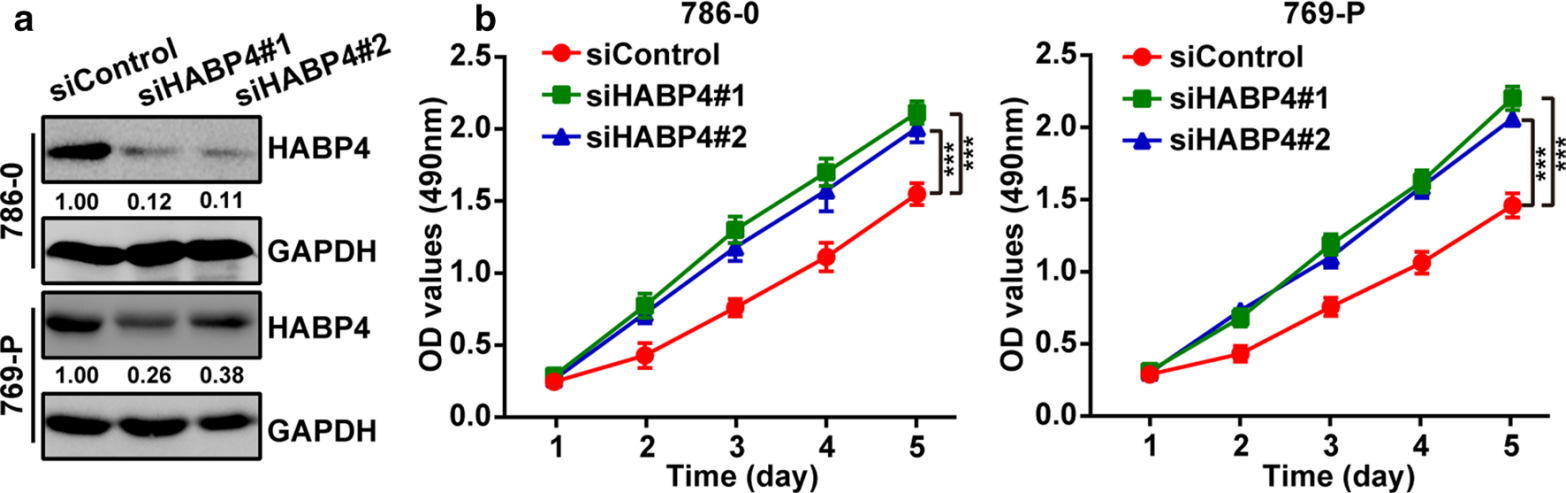
The regulatory effect of HABP4 on ccRCC cell proliferation. **a** After transfecting 786-0 and 769-P cells with two siRNAs targeting HABP4, HABP4 expression was detected using western blotting. **b** MTT assays were used to elucidate the effects of HABP4 downregulation on cell proliferation. Data are presented as mean ± SD. ***P < 0.001

**Fig. 6:**
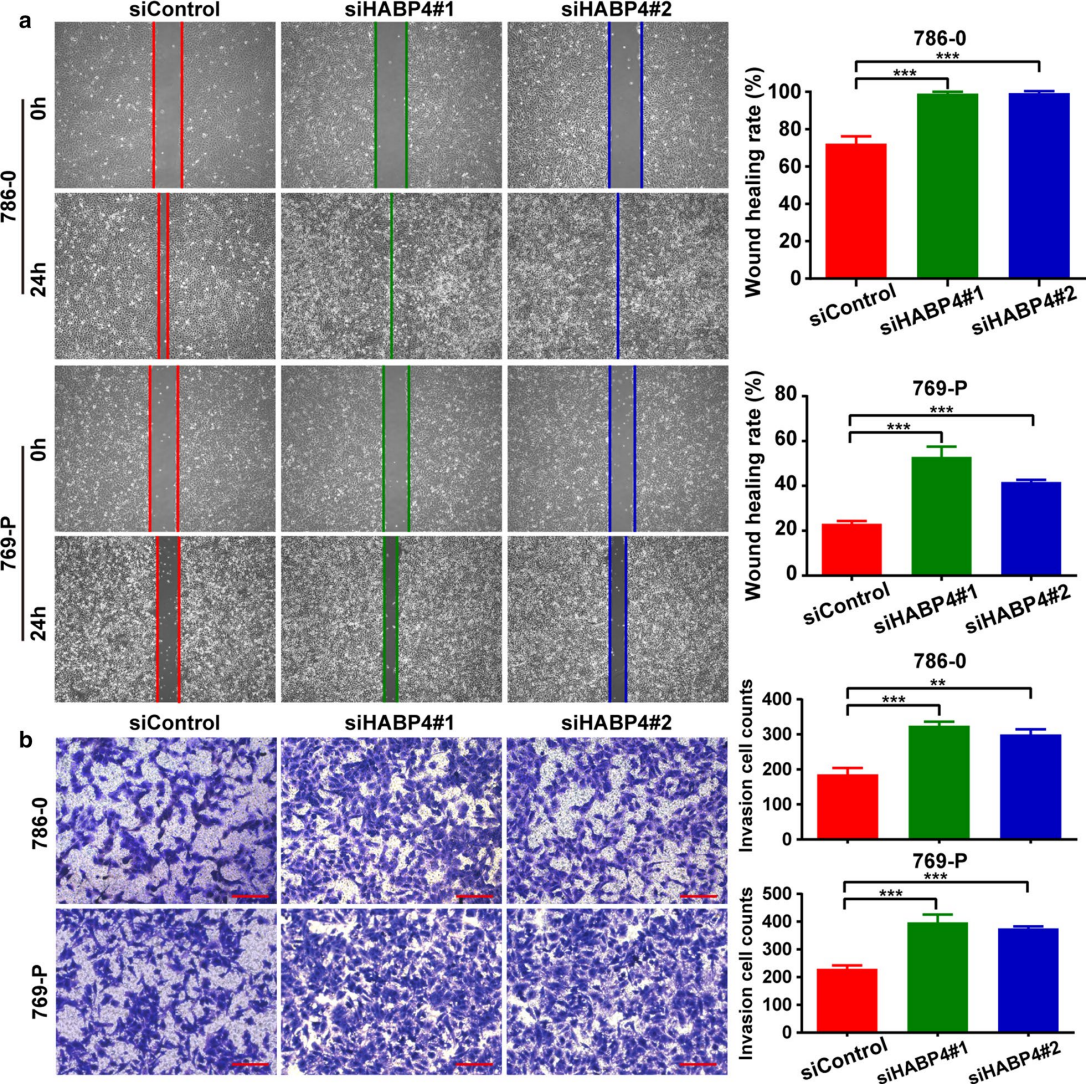
The regulatory effect of HABP4 on ccRCC cell migration and invasion. **a** Wound healing assays were used to elucidate the effects of HABP4 downregulation on cell migration. **b** Transwell invasion assays were used to analyze the role of HABP4 downregulation on cell invasion. Data are presented as mean ± SD. **P < 0.01; ***P < 0.001

To examine the effects of HABP4 on cell proliferation, migration, and invasion in ccRCC cells in vitro, HABP4 expression in cultured 796-0 and 769-P cells was downregulated using siRNA, an efficient approach used to extensively knock down specific gene expression in cells. Transfecting two siRNAs targeting HABP4 into 786-0 and 769-P cells significantly downregulated HABP4 protein expression. The two siRNAs targeting HABP4 showed nearly the same RNAi effects (Fig. 5a). MTT cell proliferation assays showed that decreased HABP4 expression could promote 786-0 and 769-P cell proliferation (Fig. 5b). Furthermore, we analyzed the effects of HABP4 on migration and invasion of ccRCC cells using wound healing analysis and transwell invasion, respectively. Accordingly, we found that reducing HABP4 expression could promote cell migration and invasion (Fig. 6a, b). The two siRNAs targeting HABP4 showed nearly the same inhibitory effects on proliferation, migration, and invasion. Thus, HABP4 appeared to have a negative regulatory effect on proliferation, migration, and invasion of ccRCC cells in vitro.

### HABP4 was a direct target of let-7i-5p

**Fig. 7:**
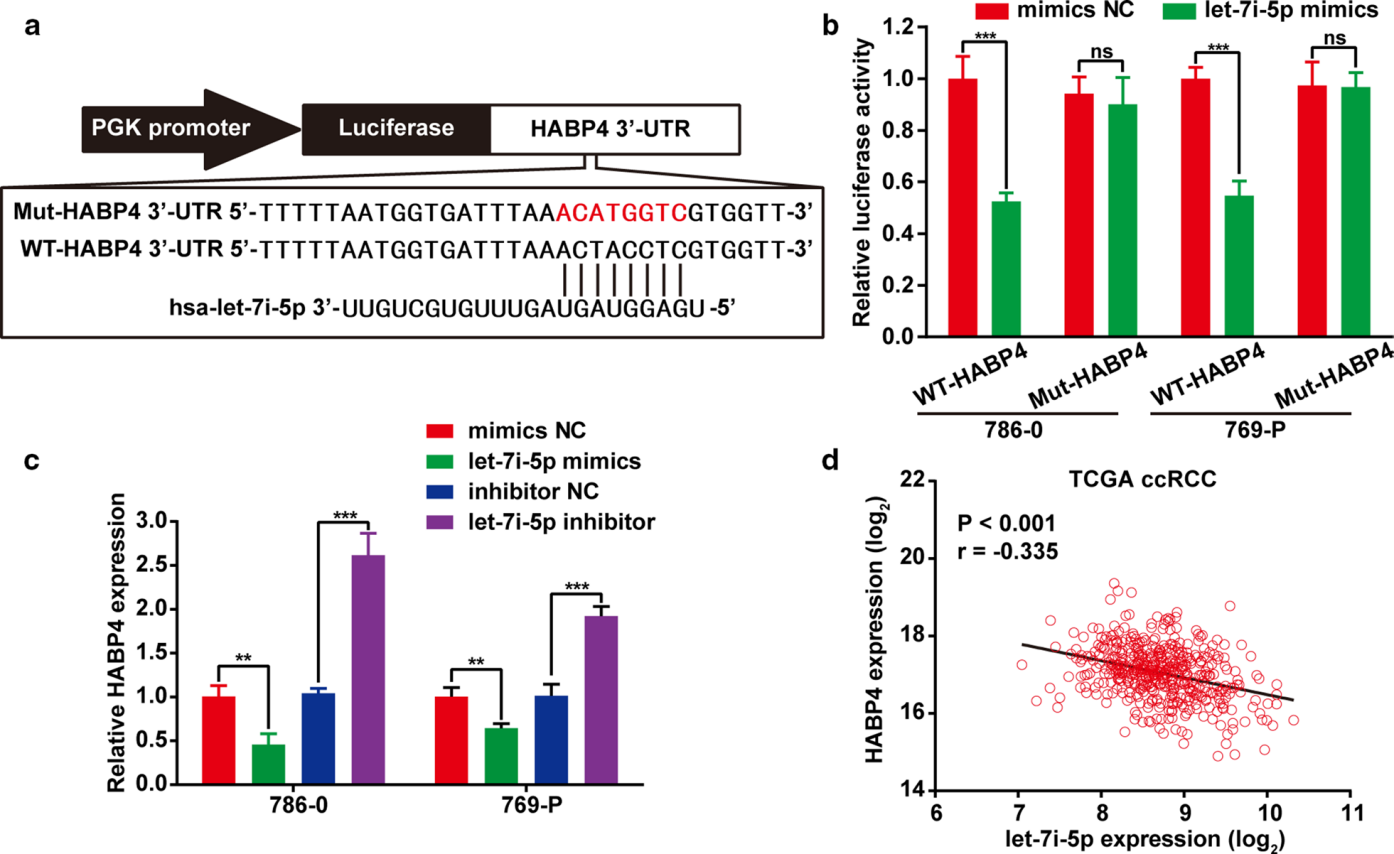
HABP4 was modulated by let-7i-5p. **a** Schematic diagram of the let-7i-5p binding sites within the 3′-UTR of HABP4 mRNA (WT-HABP4) and mutated sequences (Mut-HABP4). The mutated HABP4 3′-UTR sequence is presented in red. **b** Luciferase assay of 786-0 and 769-P cells after co-transfection with WT or Mut HABP4 3′-UTR and the let-7i-5p mimic. **c** 786-0 and 769-P cells were transfected with the let-7i-5p mimic, let-7i-5p inhibitor, and corresponding NCs and incubated for 72 h. Cells were harvested and assayed using qRT-PCR. **d** Correlation between HABP4 expression and let-7i-5p in the TCGA database. Data are presented as mean ± SD. ns > 0.05; **P < 0.01; ***P < 0.001

To determine the mechanism by which let-7i-5p regulated HABP4 expression through the HABP4 3′-UTR binding site using bioinformatics analysis, pmirGLO-WT-HABP4 and pmirGLO-MUT-HABP4 plasmids were synthesized, after which a HABP4 3′-UTR luciferase reporter assay was conducted in ccRCC cells (Fig. 7a). Accordingly, 786-0 and 769-P cells transfected with the let-7i-5p mimic and WT-HABP4 had significantly reduced luciferase activity, whereas cells transfected with MUT-HABP4 showed no reduction (Fig. 7b). To confirm the relationship between HABP4 and let-7i-5p, qRT-PCR was used to investigate the expression levels of HABP4 in ccRCC cells 72 h after transfection with the let-7i-5p mimic, miR-3175 inhibitor, or corresponding NCs. Accordingly, ccRCC cells transfected with the let-7i-5p mimic had significantly reduced HABP4 mRNA expression levels, whereas those transfected with the let-7i-5p inhibitor showed increased HABP4 mRNA expression (Fig. 7c). Meanwhile, HABP4 expression was negatively correlated with let-7i-5p in ccRCC cells (r =  − 0.335) (Fig. 7d). Taken together, our results suggested that HABP4 was a direct target of let-7i-5p.

## Discussion

The present study investigated the potential role and mechanism of let-7i-5p in ccRCC. Analysis of the TCGA database revealed that let-7i-5p expression was upregulated in ccRCC cells and that high let-7i-5p expression levels were positively correlated with poor survival. Moreover, our results showed that downregulating the expression of let-7i-5p might inhibit ccRCC cell proliferation, migration, and invasion by directly upregulating the expression of HABP4. Meanwhile, the present study has been the first to confirm that HABP4, a target gene of let-7i-5p, could be considered a tumor suppressor gene in ccRCC.

Evidence has shown that let-7i-5p, an important member of the miRNA let-7 family, played a vital role in both physiological processes and various pathological conditions [16–22], especially in tumor progression. Previous studies have shown that let-7i-5p could function as a tumor suppressor gene in various tumors, such as glioblastomas, colon cancer, gastric cancer, and esophageal carcinomas. Let-7i-5p directly targeted UDP-galactose-4-epimerase and Inhibitor of Nuclear Factor Kappa-B Kinase Subunit Epsilon to reduce glioblastoma cell proliferation and migration [11, 23]. Moreover, let-7i-5p had been found to inhibit the proliferation and metastasis of colon cancer cells by targeting kallikrein-related peptidase 6 [13] and significantly inhibit gastric cancer proliferation, invasion, and metastasis by targeting collagen type I alpha 1 chain [24]. Another study showed that let-7i-5p significantly inhibited esophageal carcinoma cell proliferation and promoted cisplatin-induced apoptosis by targeting the drug transporter ATP binding cassette subfamily C member 10 [25]. However, let-7i-5p exhibited both inhibitory and promotive functions (dual role) in the tumorigenesis of hepatocellular carcinoma. For instance, let-7i-5p had been found to not only inhibit hepatocellular carcinoma growth by targeting insulin-like growth factor 2-mRNA-binding proteins 1, 2, and 3 and the anti-apoptotic protein BCL2 like 1 [26, 27] but also rescue the tumor-suppressing effects of HDAC6 by targeting thrombospondin-1 in hepatocellular carcinoma cells [12]. Our results proved that let-7i-5p could serve to promote tumorigenesis of ccRCC.

HABP4, a nuclear and cytoplasmic regulatory protein first identified in malignant cells from Hodgkin’s lymphoma [28, 29], had been found to participate in different physiological functions, including cell proliferation, tumorigenesis, RNA transcription and splicing, and telomere maintenance. Studies have shown that HABP4 overexpression resulted in reduced cancer cell proliferation, mainly because of a G1 phase arrest [15], and that HABP4 could influence the splicing pattern of the E1A pre-mRNA by binding with the endogenous splicing proteins hnRNPQ and SFRS9 [30]. Moreover, evidence has shown that SUMOylation of HANP4 could control the formation of As_2_O_3_-induced promyelocytic leukemia nuclear bodies. Although HABP4 modification via SUMO-1 was associated with several biological processes (i.e., transcription regulation, RNA splicing, translation, ribosome biogenesis, mitotic cell cycle, and apoptotic process), SUMO-2-modified HABP4 was strongly associated with biological processes related to the control of gene expression (transcription, splicing, and translation) and telomere maintenance [31]. The present study has been the first to report that HABP4, a target gene of let-7i-5p, functioned as a tumor suppressor gene in ccRCC.

## Conclusion

The findings of the present study suggested that let-7i-5p could promote the proliferation, migration, and invasion of ccRCC cells by downregulating its target HABP4. Although we have preliminarily explored the molecular mechanism of let-7i-5p regulating the proliferation, migration, and invasion of ccRCC cells in vitro, further in vivo experiments are needed to verify the feasibility of let-7i-5p as a potential therapeutic target for ccRCC in future studies.

## Supplementary Information

**Additional file 1:** Supplementary Table S1: The let‑7i‑5p expression data and clinical information of ccRCC patients.

**Additional file 2:** Supplementary Table S2: 234 genes that negatively correlated with let-7i-5p expression.

## Data Availability

The data and materials of this experiment are available.

## Abbreviations

ccRCC: Clear cell renal cell carcinoma
RCC: Renal cell carcinoma
nccRCC: Non-clear cell renal cell carcinoma
miRNAs: MicroRNAs
mRNA: Messenger RNA
HABP4: Hyaluronan-binding protein 4
qRT-PCR: Quantitative real-time PCR
DMEM: Dulbecco’s Modified Eagle’s Medium
