# Supplementary material for: Let-7i-5p enhances cell proliferation, migration and invasion of ccRCC by targeting HABP4
Source: BMC Urol. 2021 Mar 28;21:49. doi: 10.1186/s12894-021-00820-9 (PMC8005230; doi:10.1186/s12894-021-00820-9)
Supplement: Supplementary file 2 — Additional file 2: Supplementary Table S2: 234 genes that negatively correlated with let-7i-5p expression. [file 12894_2021_820_MOESM2_ESM.docx]

**Additional file 1: Table S1:** 234 genes that negatively correlated with let-7i-5p expression

|  | | | | | | | | |
| --- | --- | --- | --- | --- | --- | --- | --- | --- |
| Gene Symbol | Pearson's r | P-value | Gene Symbol | Pearson's r | P-value | Gene Symbol | Pearson's r | P-value |
| ABCA5 | -0.3274 | 9.29E-08 | FAM47E | -0.3883 | 1.44E-10 | PLRG1 | -0.3334 | 5.19E-08 |
| ACAA2 | -0.3322 | 5.86E-08 | FAM54B | -0.3190 | 2.04E-07 | PNPLA7 | -0.3038 | 8E-07 |
| ACAT1 | -0.3205 | 1.79E-07 | FANCC | -0.3816 | 3.15E-10 | PPARG | -0.3191 | 2.03E-07 |
| ADHFE1 | -0.3297 | 7.46E-08 | FBXO9 | -0.3540 | 6.52E-09 | PPARGC1A | -0.3022 | 9.18E-07 |
| AGAP11 | -0.3098 | 4.73E-07 | FLJ11235 | -0.3577 | 4.4E-09 | PPP1R13B | -0.3303 | 7.02E-08 |
| AGPAT9 | -0.3145 | 3.09E-07 | FLJ13197 | -0.3334 | 5.22E-08 | PRR5-ARHGAP8 | -0.3092 | 4.98E-07 |
| AGTR1 | -0.3228 | 1.44E-07 | FRMD1 | -0.3600 | 3.44E-09 | PTH1R | -0.3643 | 2.16E-09 |
| AKD1 | -0.3106 | 4.38E-07 | GARNL3 | -0.3875 | 1.59E-10 | PTPN3 | -0.3806 | 3.51E-10 |
| AKR7L | -0.3035 | 8.25E-07 | GBA2 | -0.3806 | 3.52E-10 | RAB3IP | -0.3650 | 2.01E-09 |
| AMY2B | -0.3010 | 1.02E-06 | GLS2 | -0.3653 | 1.95E-09 | RABGAP1 | -0.3403 | 2.65E-08 |
| ANAPC2 | -0.3009 | 1.03E-06 | GPR17 | -0.3310 | 6.56E-08 | RALGPS1 | -0.3292 | 7.8E-08 |
| ANKRD56 | -0.3352 | 4.39E-08 | GPRASP1 | -0.3365 | 3.86E-08 | RAPGEF2 | -0.3271 | 9.59E-08 |
| ANKS6 | -0.3103 | 4.51E-07 | GRAMD1C | -0.3159 | 2.72E-07 | RAPGEF3 | -0.3202 | 1.83E-07 |
| AQP1 | -0.3019 | 9.44E-07 | GTF2IRD2 | -0.3012 | 1.01E-06 | RBPMS2 | -0.3321 | 5.94E-08 |
| ARHGAP24 | -0.3510 | 8.88E-09 | GTF2IRD2P1 | -0.3163 | 2.61E-07 | RG9MTD3 | -0.3432 | 1.98E-08 |
| ARHGAP8 | -0.4001 | 3.51E-11 | HABP4 | -0.3323 | 5.82E-08 | RGL3 | -0.3825 | 2.85E-10 |
| ARHGEF9 | -0.3056 | 6.85E-07 | HIBCH | -0.3654 | 1.94E-09 | RNPC3 | -0.3308 | 6.71E-08 |
| ASTN2 | -0.3265 | 1.01E-07 | HLF | -0.3609 | 3.12E-09 | SCN4A | -0.3033 | 8.41E-07 |
| AUH | -0.3715 | 9.84E-10 | HOOK2 | -0.3472 | 1.31E-08 | SECISBP2 | -0.3554 | 5.65E-09 |
| BAG1 | -0.3245 | 1.23E-07 | HSD11B2 | -0.3311 | 6.51E-08 | SENP8 | -0.3057 | 6.8E-07 |
| BBS1 | -0.3777 | 4.9E-10 | HSD17B8 | -0.3416 | 2.32E-08 | SGSM1 | -0.3529 | 7.3E-09 |
| BCKDHB | -0.3053 | 7.04E-07 | HSDL2 | -0.3260 | 1.06E-07 | SIRT5 | -0.3700 | 1.17E-09 |
| BDH2 | -0.3371 | 3.64E-08 | IKBKAP | -0.3175 | 2.34E-07 | SLC16A11 | -0.3175 | 2.34E-07 |
| BDNFOS | -0.3417 | 2.29E-08 | IL17RD | -0.3637 | 2.32E-09 | SLC22A13 | -0.3243 | 1.25E-07 |
| BRP44L | -0.3385 | 3.16E-08 | IMPA2 | -0.3626 | 2.62E-09 | SLC25A4 | -0.3758 | 6.06E-10 |
| BSPRY | -0.3219 | 1.56E-07 | IMPG1 | -0.3004 | 1.08E-06 | SLC2A11 | -0.3630 | 2.5E-09 |
| BTBD9 | -0.3175 | 2.35E-07 | INTU | -0.3276 | 9.11E-08 | SLC2A4 | -0.3853 | 2.04E-10 |
| BTNL9 | -0.3295 | 7.57E-08 | IVNS1ABP | -0.3412 | 2.42E-08 | SLC5A8 | -0.3367 | 3.78E-08 |
| C16orf3 | -0.3302 | 7.12E-08 | KANK3 | -0.3240 | 1.29E-07 | SNORD116-28 | -0.3050 | 7.23E-07 |
| C1orf203 | -0.3468 | 1.38E-08 | KAT5 | -0.3415 | 2.33E-08 | SPATA24 | -0.3541 | 6.48E-09 |
| C1orf210 | -0.3911 | 1.03E-10 | KBTBD3 | -0.3140 | 3.23E-07 | STX17 | -0.3232 | 1.38E-07 |
| C5orf23 | -0.3137 | 3.31E-07 | KIAA0649 | -0.3546 | 6.15E-09 | SUGT1L1 | -0.3494 | 1.04E-08 |
| C6orf124 | -0.3309 | 6.66E-08 | KIAA0664P3 | -0.4447 | 9.79E-14 | SYN3 | -0.3320 | 5.99E-08 |
| C6orf155 | -0.4261 | 1.27E-12 | KIAA1328 | -0.3223 | 1.5E-07 | TBC1D13 | -0.3271 | 9.52E-08 |
| C6orf164 | -0.3704 | 1.12E-09 | KIAA1529 | -0.3489 | 1.1E-08 | TEF | -0.4269 | 1.14E-12 |
| C6orf217 | -0.3260 | 1.06E-07 | KIF12 | -0.3397 | 2.82E-08 | THSD1P1 | -0.3070 | 6.02E-07 |
| C7orf41 | -0.3676 | 1.52E-09 | KRBA2 | -0.3431 | 2E-08 | TJP2 | -0.3247 | 1.2E-07 |
| C9orf106 | -0.3408 | 2.5E-08 | LDHD | -0.3296 | 7.55E-08 | TMEM80 | -0.3606 | 3.24E-09 |
| C9orf125 | -0.3075 | 5.77E-07 | LIN7A | -0.3169 | 2.47E-07 | TMEM8B | -0.3501 | 9.8E-09 |
| C9orf130 | -0.3198 | 1.9E-07 | LLGL2 | -0.3267 | 9.89E-08 | TPPP | -0.3134 | 3.41E-07 |
| C9orf156 | -0.4083 | 1.27E-11 | LOC100190938 | -0.3142 | 3.16E-07 | TSC1 | -0.4487 | 5.52E-14 |
| C9orf3 | -0.3263 | 1.04E-07 | LOC100289341 | -0.3523 | 7.77E-09 | TSPAN7 | -0.3467 | 1.39E-08 |
| C9orf68 | -0.3271 | 9.53E-08 | LOC147727 | -0.3465 | 1.41E-08 | TUB | -0.3047 | 7.41E-07 |
| CACNB2 | -0.3040 | 7.89E-07 | LOC284440 | -0.3060 | 6.59E-07 | UNC13B | -0.3543 | 6.34E-09 |
| CALCOCO1 | -0.3284 | 8.46E-08 | LOC286367 | -0.3383 | 3.22E-08 | VPS13A | -0.3179 | 2.27E-07 |
| CAPN13 | -0.3028 | 8.75E-07 | LOC388387 | -0.3657 | 1.87E-09 | WLS | -0.3091 | 5.02E-07 |
| CARKD | -0.3095 | 4.86E-07 | LOC646471 | -0.3145 | 3.08E-07 | XPA | -0.3330 | 5.44E-08 |
| CBARA1 | -0.3112 | 4.16E-07 | LOC91316 | -0.3213 | 1.65E-07 | YTHDC1 | -0.3015 | 9.83E-07 |
| CBR4 | -0.3160 | 2.69E-07 | MAP7 | -0.3243 | 1.25E-07 | ZADH2 | -0.3408 | 2.51E-08 |
| CBX7 | -0.3399 | 2.75E-08 | MCF2 | -0.3052 | 7.06E-07 | ZBTB3 | -0.3055 | 6.88E-07 |
| CDADC1 | -0.3598 | 3.52E-09 | MCF2L | -0.3407 | 2.53E-08 | ZBTB5 | -0.3550 | 5.87E-09 |
| CDC14B | -0.3403 | 2.65E-08 | MED22 | -0.3255 | 1.12E-07 | ZDHHC6 | -0.3110 | 4.23E-07 |
| CDK20 | -0.3381 | 3.3E-08 | MRPL50 | -0.3423 | 2.16E-08 | ZER1 | -0.3510 | 8.95E-09 |
| CDS1 | -0.3393 | 2.93E-08 | MRPS31 | -0.3366 | 3.82E-08 | ZNF132 | -0.3909 | 1.06E-10 |
| CENPV | -0.3422 | 2.19E-08 | MRPS9 | -0.3023 | 9.14E-07 | ZNF135 | -0.3247 | 1.2E-07 |
| CGN | -0.3376 | 3.46E-08 | MRRF | -0.3437 | 1.87E-08 | ZNF154 | -0.3233 | 1.37E-07 |
| CHAD | -0.3226 | 1.46E-07 | MUC20 | -0.3324 | 5.76E-08 | ZNF204P | -0.3645 | 2.13E-09 |
| CIRBP | -0.3219 | 1.56E-07 | MUC4 | -0.3128 | 3.59E-07 | ZNF211 | -0.3158 | 2.75E-07 |
| CPEB3 | -0.3019 | 9.47E-07 | MYL3 | -0.3916 | 9.73E-11 | ZNF274 | -0.3119 | 3.92E-07 |
| CPO | -0.3514 | 8.51E-09 | NDRG2 | -0.3377 | 3.43E-08 | ZNF304 | -0.3064 | 6.37E-07 |
| CRAT | -0.3076 | 5.74E-07 | NEIL1 | -0.3031 | 8.55E-07 | ZNF397 | -0.3062 | 6.48E-07 |
| CRY2 | -0.4516 | 3.63E-14 | NICN1 | -0.3880 | 1.49E-10 | ZNF418 | -0.3326 | 5.64E-08 |
| DET1 | -0.3125 | 3.71E-07 | NINL | -0.3169 | 2.48E-07 | ZNF433 | -0.3663 | 1.74E-09 |
| DMRTA1 | -0.3122 | 3.8E-07 | NIPSNAP3B | -0.3052 | 7.09E-07 | ZNF471 | -0.3485 | 1.15E-08 |
| DNAJC28 | -0.3225 | 1.48E-07 | NOXA1 | -0.3374 | 3.54E-08 | ZNF483 | -0.3116 | 4.01E-07 |
| DZIP3 | -0.3205 | 1.78E-07 | NR3C2 | -0.3336 | 5.13E-08 | ZNF540 | -0.3927 | 8.53E-11 |
| EFCAB6 | -0.3052 | 7.09E-07 | NUDT6 | -0.3221 | 1.53E-07 | ZNF549 | -0.3557 | 5.46E-09 |
| EMX2OS | -0.3150 | 2.96E-07 | OGDHL | -0.3044 | 7.58E-07 | ZNF577 | -0.3170 | 2.45E-07 |
| ENAM | -0.3845 | 2.25E-10 | OTUD7A | -0.3180 | 2.25E-07 | ZNF594 | -0.3091 | 5E-07 |
| ENOSF1 | -0.3117 | 3.98E-07 | OVOL1 | -0.3717 | 9.63E-10 | ZNF671 | -0.3821 | 2.97E-10 |
| ENPP5 | -0.3011 | 1.01E-06 | PCCA | -0.4030 | 2.44E-11 | ZNF76 | -0.3748 | 6.79E-10 |
| EPB41L5 | -0.3075 | 5.77E-07 | PER3 | -0.3791 | 4.2E-10 | ZNF763 | -0.3727 | 8.61E-10 |
| EPHX2 | -0.3563 | 5.12E-09 | PET112L | -0.3388 | 3.08E-08 | ZNF773 | -0.3315 | 6.27E-08 |
| ERBB2 | -0.3581 | 4.24E-09 | PKHD1 | -0.3350 | 4.46E-08 | ZNF780B | -0.3465 | 1.42E-08 |
| ETFDH | -0.3354 | 4.31E-08 | PLEKHA7 | -0.3101 | 4.58E-07 | ZNF782 | -0.3536 | 6.79E-09 |
| EZH1 | -0.3555 | 5.58E-09 | PLEKHH1 | -0.3546 | 6.13E-09 | ZNF833 | -0.3138 | 3.28E-07 |
| FAAH | -0.3096 | 4.8E-07 | PLEKHH2 | -0.3024 | 9.03E-07 | ZNF839 | -0.3325 | 5.69E-08 |
| FAM160A1 | -0.3181 | 2.23E-07 | PLLP | -0.3331 | 5.34E-08 | ZSCAN18 | -0.4155 | 5.08E-12 |
